# Eco-Friendly Supercapacitors Based on Biodegradable Poly(3-Hydroxy-Butyrate) and Ionic Liquids

**DOI:** 10.3390/nano10102062

**Published:** 2020-10-19

**Authors:** Lorenzo Migliorini, Tommaso Santaniello, Francesca Borghi, Paolo Saettone, Mauro Comes Franchini, Gianluca Generali, Paolo Milani

**Affiliations:** 1 Interdisciplinary Centre for Nanostructured Materials and Interfaces (CIMaINa), Physics Department, University of Milan, 20133 Milano, Italy; lorenzo.migliorini@unimi.it (L.M.); tommaso.santaniello@unimi.it (T.S.); francesca.borghi@unimi.it (F.B.); 2 Bio-On spa, Via Santa Margherita al Colle 10/3, 40136 Bologna, Italy; Paolosaettone@yahoo.it (P.S.); gianluca.generali@bio-on.it (G.G.); 3 Department of Industrial Chemistry “Toso Montanari”, University of Bologna, Viale Risorgimento 4, 40136 Bologna, Italy

**Keywords:** green electronics, supercapacitors, polyhydroxyalkanoates, bioplastic, ionic liquids, supersonic cluster beam deposition, EDLC

## Abstract

The interest for biodegradable electronic devices is rapidly increasing for application in the field of wearable electronics, precision agriculture, biomedicine, and environmental monitoring. Energy storage devices integrated on polymeric substrates are of particular interest to enable the large-scale on field use of complex devices. This work presents a novel class of eco-friendly supercapacitors based on biodegradable poly(3-hydroxybutyrrate) PHB, ionic liquids, and cluster-assembled gold electrodes. By electrochemical characterization, we demonstrate the possibility of tuning the supercapacitor energetic performance according to the type and amount of the ionic liquid employed. Our devices based on hydrophobic plastic materials are stable under cyclic operation and resistant to moisture exposure.

## 1. Introduction

Natural-derived and biodegradable resources and raw materials (such as cellulose or bioplastics) are increasingly used as building blocks for the development of biodegradable electronics and robotics [1,2,3,4] in fields such as precision agriculture [5], wearable electronics [6,7], biomedicine [8], and environmental monitoring [9]. A critical aspect for these applications is represented by energy autonomy, that is the capability of remotely operating and communicating in a continuous mode without a physical connection to a physical network.

Electrolytic double-layer capacitors (EDLCs) [10] are a particularly performing class of supercapacitors (SCs) [11,12,13]; they are usually composed by a liquid or semi-solid electrolyte sandwiched between (or connecting) a couple of high-surface area electrodes. By the application of an electric potential difference, the ions of the electrolyte migrate towards the electrodes with an opposite charge, forming an electrolytic double-layer at the electrode-electrolyte interface. This process is reversible and supercapacitors can undergo a high number of charge-discharge cycles. The maximum work potential is limited by the electrochemical stability of the electrolyte (usually between 1 and 4 Volt) [14]. Carbon-derived porous materials are the most employed for the fabrication of EDLCs electrodes since they combine lightness and high surface area. Activated carbon and carbon fibres, graphene, single-walled, and multi-walled carbon nanotubes are among the most employed [15,16,17,18,19,20,21], even if they sometimes present a low electronic conductance and environmental issues [22]. The lightness of carbon electrodes allows the fabrication of devices characterized by high values of gravimetric stored energy and delivery power. Nevertheless, their porous structure results in a large occupied volume, and in recent years, volumetric performances are becoming more and more crucial for the realization of more compact mobile and portable electrical systems and devices. The work of Li et al. [23] highlights these aspects and encourages the use of small volume electrodes for supercapacitors. Concerning the liquid electrolyte media, the most employed are aqueous and organic: the first have a high ionic conductivity and a low operating voltage [24], while the second possess a wider electrochemical window of stability, but lower ionic conductivity and high toxicity. Both evaporate in ambient conditions and are sensitive to environmental humidity, making mandatory a protection and/or encapsulation [16]. Ionic liquids (ILs) can also be employed as electrolytic medium [14,15,25]. Despite a lower ionic conductivity, their low vapour pressure makes them stable in ambient conditions, their electrochemical window can reach 4 Volt, and their cytotoxicity is extremely low compared to that of traditional organic solvents [26,27]. In recent years, most EDLCs have been developed with the use of various polymeric matrices blended with liquid electrolytes, resulting in solid films of gel in which the (solvated) ions are still able to migrate. The all-solid structure avoids the problem of leakage and is more suited for applications in micro-, soft, and deformable electronics [28]. Gel electrolytes can be obtained with different techniques using various combinations of polymers and electrolytes. Water containing gels are called hydrogels [29], while ionogels are obtained with ionic liquids [30]. A three-dimensional polymeric matrix can be achieved with chemical bonds between different polymeric chains (chemical gels) but also by exploiting low-strength inter-chains interactions (physical gels) such as dipole-dipole or Van del Waals interactions [31]. The combination with a polymer typically decreases the ionic conductivity of the electrolyte, but it is a drawback that is worth to pay in order to obtain a system that can be integrated in a soft and deformable device.

To face the challenge of environmental sustainability, in recent years many researchers focused on the use of natural-derived and biodegradable polymers as building blocks for EDLCs. Carbohydrates are the most employed for this purpose, cellulose on top; an exhaustive review on the topic is given by Zhang et al. [32]. For example, carbon electrodes have been obtained from natural lignin or wood [33,34,35], while other works reported biodegradable electrolyte gels from cellulose derivatives, agarose, or silicates [36,37,38]. Nevertheless, these supercapacitors absorb the surrounding humidity and their energetic performance are influenced by the environmental moisture; for this reason, they need to be protected through encapsulation or sealing. Moisture resistant electrolyte gels would be highly desirable for the realization of supercapacitors able to stably operate in different environments without any encapsulation. Among natural polymers, poly(3-hydroxybutyrrate) (PHB) is a polyester produced by bacteria and readily biodegradable [39,40,41,42], never employed before for the development of supercapacitors. As typical of bioplastic materials, PHB is highly hydrophobic and it can represent a promising choice for the realization of moisture resistant EDLC supercapacitors.

In this work, we report the synthesis and the electrochemical characterization of moisture resistant supercapacitors (EDLC type) based on PHB bioplastic. PHB-based ionogels are obtained by blending PHB powder with three different kinds of ionic liquids: 1-butyl-3-methylimidazolium bis(trifluoromethylsulfonyl)imide BMIM(TFSI), 1-ethyl-3-methylimidazolium bis(trifluoromethylsulfonyl)imide EMIM(TFSI), and choline bis(trifluoromethylsulfonyl)imide Chol(TFSI). Gold electrodes are deposited on the surface of the ionogels by means of supersonic cluster beam deposition (SCBD) [43,44,45,46,47,48,49,50]. The electrochemical characterization highlights the energetic behaviour of the supercapacitors and shows how the type and the amount of the employed ionic liquid can affect the resulting properties. The influence of the surrounding humidity was also studied by testing the supercapacitors in ambient conditions, as well as in inert nitrogen atmosphere.

## 2. Materials and Methods

### 2.1. Materials and Reagents

Purified poly(3-hydroxybutyrate) (PHB) powder was provided by the company Bio-On Spa. (Via Santa Margherita Al Colle, 10/3, 40136 Bologna BO, Italy). Ionic liquids 1-ethyl-3-methylimidazolium bis(trifluoromethylsulfonyl)imide 99% (EMIM(TFSI)), 1-buthyl-3-methylimidazolium bis(trifluoromethylsulfonyl)imide 99% (BMIM(TFSI)), and choline bis(trifluoromethylsulfonyl)imide 99% (Chol(TFSI)) were purchased from Iolitec GmbH (Salzstraße 184, 74076 Heilbronn, Germany). Tetrabuthylammonium fluoride hydrate 98% (TBAF) was purchased from Sigma Aldrich (Via Gallarate, 154, 20151 Milano MI, Italy) and glacial acetic acid from Riedel-de Haen (Charlotte, North Carolina, NC, US). Gold rods 99.9% were purchased from 8853 Spa (Via Pitagora, 11, 20016 Pero MI, Italy).

### 2.2. Ionogel Synthesis

**Figure 1:**
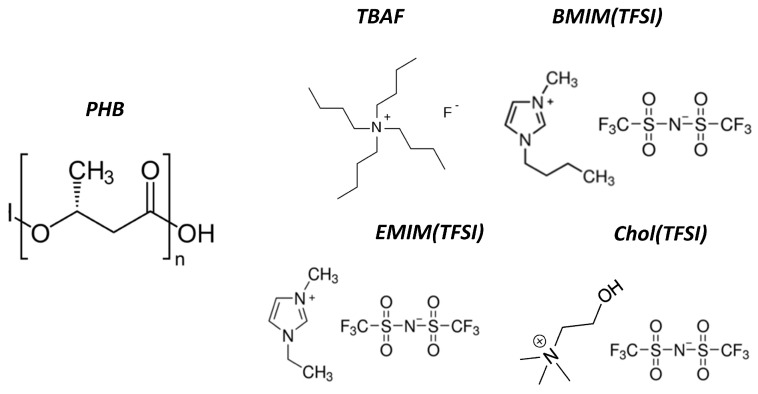
The chemical structure of the polymeric matrix is shown on the left side of the picture, while the other additives (ILs and organic salt) are shown on the right side.

Figure 1 reports the chemical structures of the employed reagents. Ionic mixtures were prepared by dissolving TBAF in the proper ionic liquid at a concentration of 6.85% *w*/*w* and keeping it under magnetic stirring for 24 h. PHB powder was dissolved in acetic acid (50 mg/mL) preheated at 110 °C with the help of an oil bath and a heating plate. After 8 min of magnetic stirring, different amounts of ionic mixtures were also added, from 1.1 to 2.9 *w*/*w* with respect to PHB mass. After another 7 min of magnetic stirring, the solution was poured into a preheated (for 30 min) aluminum mold over a surface area of 70 × 25 mm^2^ and stored in an oven at 105 °C. After 30 min, a 50 μm-thick solid film formed and was then peeled off. Some ionogels were also synthesized without the use of TBAF salt to have a comparison (in fact, TBAF provides additional ions that can affect the formation of the electrolytic double-layer. The obtained samples were kept in vacuum for at least 16 h to remove any residual of acetic acid. The ionogels obtained with the BMIM(TFSI), EMIM(TFSI), and Chol(TFSI) ionic liquids will be noted as BT, ET, and CT, respectively. The abbreviations of each sample will contain the corresponding w/w ratio as well (e.g., the sample BT1.7 is the one with BMIM(TFSI) and a mass ratio of 1.7 between the ionic liquid and PHB). A detailed table of all the ionogels formulation can be found in the Supplementary Materials (Table S1).

The weight of the ionogels (m_0_) was measured after drying the material in vacuum. To investigate on their moisture absorption, samples were put inside an incubator (Galaxy S, RSBiotech, provided by Samson Scientific, 1 Simonsburn Road, Loreny Industrial Estate, Kilmarnock, Ayrshire, Scotland) at 37 °C and a humidity level of 95% for 24 h. Their weight was then measured again (m_f_) and the moisture absorption was estimated as the difference between m_f_ and m_0_.

### 2.3. Electrode Deposition by Supersonic Cluster Beam Deposition (SCBD)

**Figure 2:**
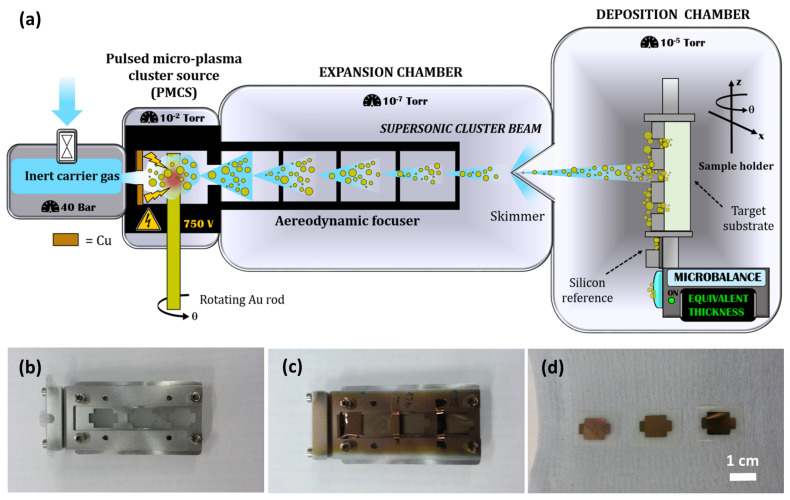
(**a**) Schematic representation of the Supersonic cluster beam deposition (SCBD) apparatus (not to scale) and its working principle; (**b**) ionogel samples mounted on the sample holder together with the stencil masks, before and (**c**) after the deposition process; (**d**) examples of the obtained PHB-IL-Au supercapacitors.

Cluster-assembled gold electrodes were fabricated on both sides of the ionogels using a Supersonic cluster beam deposition (SCBD) apparatus equipped with a pulsed microplasma cluster source (PMCS) [51] (see Figure 2a). Gold clusters produced in the PMCS are seeded in supersonic expansion of a carrier inert gas (Ar), pass through an aerodynamic focuser [52], and impinge the surface of the ionogel. The ionogel samples were mounted on a custom-designed sample holder (shown in Figure 2b,c) inside the deposition chamber. Stencil masks were used to obtain electrodes of 0.8 × 0.8 cm^2^. A coaxial quartz microbalance was also targeted by the cluster beam in order to measure in real time the deposited Au mass, which is correlated to the equivalent thickness t_eq_ of the deposited layer, defined as the thickness that such layer should have if deposited on a smooth and rigid substrate. t_eq_ was also checked afterwards with a profilometer (KLA Tencor P-6) on a silicon wafer targeted as well by the cluster beam. Knowing both the thickness and the mass of the deposited Au layers allowed to calculate its density as well. The average Au deposition rate was kept at a value of ca. 0.5 nm/s until a final t_eq_ = 150 nm was reached. During all the processes, the pressure in the expansion and deposition chamber were about 1.0 × 10^−7^ Torr and 1.0 × 10^−5^ Torr, respectively. Figure 2d shows a picture of the resulting PHB-IL-Au supercapacitors.

### 2.4. Morphological Characterization

Scanning electron microscopy (SEM) imaging was performed on the metallized samples using a FEG-scanning electron microscopy, SEM model Zeiss Supra 40. The morphology of the ionogels was characterized by atomic force microscopy (AFM) using a Bioscope Catalyst (Bruker) instrument, operated in Peak-Force Tapping Mode in air. From flattened AFM images, the root-mean-square surface roughness *R_q_* was calculated as the standard deviation of surface heights. More details are reported in the Appendix A: experimental details on AFM measurements.

### 2.5. Electrochemical Characterization

The electrochemical analysis on the ionogel-Au composites were carried out in a two-electrodes setup using a Gamry potentiostat (model Reference 600) and a Gamry software, in ambient conditions and inside a glove box in inert N_2_ atmosphere (oxygen concentration equal to 0.03 ppm). Soft contacts were employed to connect the samples to the instrument. A schematic representation of the employed setup is provided in the Supplementary Materials (Figure S1). Electrochemical impedance spectroscopy (EIS) was carried out as follows: an AC = 5 mV was superimposed at a DC = 0 V and the scanned frequency range f was between 1 × 10^−2^ Hz and 1 × 10^6^ Hz. The equivalent series resistance (ESR) was obtained from the Nyquist plot by considering the intercept with the X-axis. The double-layer capacitance *C_dl_* was calculated according Equation (1):

(1) $$C_{dl}\left(f\right)=\frac{-Z_{i}\left(f\right)}{2\pi f{\left|Z\left(f\right)\right|}^{2}\;\times \;A}$$

where *Z_i_* is the imaginary component of the impedance, |*Z*| its modulus, and *A* is the area of the gold electrodes. Cyclic voltammetry was performed using a potential range between −2.0 V and +2.0 V at a scanning rate of 10 mV/s. Galvanostatic Charge and Discharge (GCD) was conducted by charging the supercapacitors at ∆*V_max_* values of 1.0 and 1.5 V with current densities *i* equals to 4, 8, 16, 32, 64, 128, and 256 μA/cm^2^. The coulombic efficiency CE was calculated as the ratio between the time of discharge *t_d_* and the time of charge *t_c_*. The capacitance and equivalent series resistance were calculated according Equations (2) and (3):

(2) $$C_{dl}\left[\frac{F}{{\mathrm{cm}}^{2}}\right]=\;\frac{i\;\left[\frac{A}{{\mathrm{cm}}^{2}}\right]\;\times \;t_{d}\;\left[s\right]}{\Delta V_{max}\;\left[V\right]}$$

(3) $$\mathrm{ESR}\;\left[\Omega \;\times {\;\mathrm{cm}}^{2}\right]=\;\frac{\Delta U\;\left[V\right]}{2i\;\left[\frac{A}{{\mathrm{cm}}^{2}}\right]}$$

where ∆*U* is the potential drop at the beginning of the discharge [53,54]. Cyclic GCD tests were also carried out, up to a cycle number of 10^4^. The capacitance retention *C_ret_* (%) was calculated as the ratio between *C_n_* and *C*_0_, where *C*_0_ is the capacitance during the 3rd cycle and *C_n_* is the capacitance value obtained at a generic nth cycle. The volumetric stored energy E*_V_* and average delivery power P*_V_* were calculated according to Equations (4) and (5) [55]:

(4) $$E_{V}\;\left[\mathrm{Wh}/{\mathrm{dm}}^{3}\right]=\;\frac{\int_{0}^{t_{d}}i\;\left[\frac{A}{{\mathrm{cm}}^{2}}\right]\;\times \;\Delta V\;\left[V\right]\;dt}{3600\;\times \;V_{E}\;\left[{\mathrm{dm}}^{3}\right]}$$

(5) $$P_{V}\;\left[W/{\mathrm{dm}}^{3}\right]=\;\frac{3600\;\times \;E_{V}\;\left[\mathrm{Wh}/{\mathrm{dm}}^{3}\right]}{t_{d}\;\left[s\right]}$$

where *V_E_* is the electrodes’ volume. 

## 3. Results and Discussion

### 3.1. Supercapacitors Fabrication

Thin ionogel films were obtained through a simple process of solvent casting from acetic acid, blending together PHB powder and three different kinds of ionic liquids: BMIM(TFSI), EMIM(TFSI), and Chol(TFSI). They were chosen according to the empirical observation of good miscibility between PHB and TFSI-anion ionic liquids. Among them, Chol(TFSI) possess the biomolecule choline as cation and it represents an interesting ingredient in view of a full biodegradable supercapacitor. Different relative amounts of ionic liquid and the inclusion of TBAF were investigated in order to study the effect on the electrochemical properties and energetic performance of the resulting supercapacitors. The moisture influence was checked after conditioning the samples for 24 h in an incubator at 37 °C with humidity level of 95%. A small increase of about 1–2% of the original mass was observed only for samples with a higher amount of IL. These bioplastic-based ionogels can then be considered unaffected by environmental humidity. Each ionogel sample was then provided with a couple of 150 nm-thick cluster-assembled Au electrodes (in a sandwich geometry) by means of supersonic cluster beam deposition (SCBD) [43,44,45,46,47,48,49,50]. Cluster-assembled Au electrodes have a granular structure characterized by a high electrical conductivity typical of gold and a low density of about 8.0 g/cm^3^ [56]. It was already demonstrated that supersonically accelerated Au clusters can partially penetrate and implant into the surface of soft polymeric substrates, leading to a high interfacial area and robust adhesion [52,57], providing an efficient and stable electrolytic double layer at the electrode-ionogel interface. Beside this, the SCBD technique is an eco-friendly solvent-free technique that works at room temperature, a great advantage while dealing with thermolabile natural polymers.

**Figure 3:**
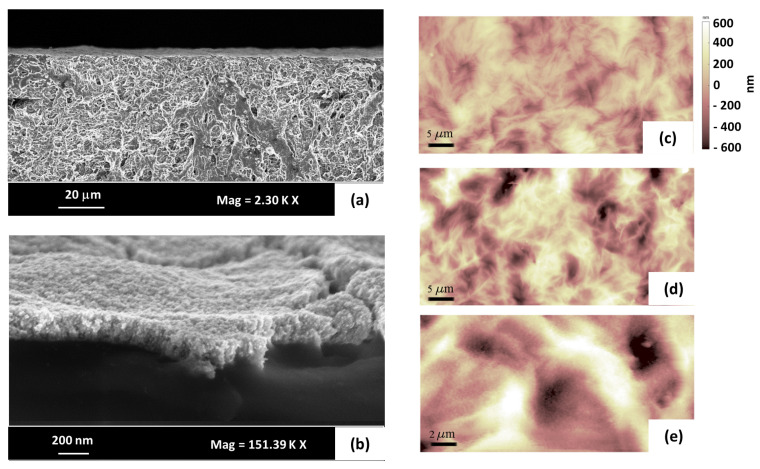
(**a**) Scanning electron microscopy (SEM) image showing the cross-section of supercapacitor BT1.7; (**b**) SEM image focusing on the electrodes cross-section; (**c**,**d**) atomic force microscopy (AFM) images of the ionogels surfaces BT1.7 and BT 2.9 (respectively); (**e**) AFM image of the gold surface deposited on top of the ionogel BT1.7.

Imaging of the samples BT1.7 and BT2.9 was also carried out by mean of SEM and AFM techniques. SEM images are reported in Figure 3a,b, showing the ionogels cross-section as well as the cluster-assembled structure of the electrodes. AFM measurements detected an average roughness of 125 ± 27 nm (BT1.7) (Figure 3c) and 150 ± 26 nm (BT2.9) (Figure 3d) on the surface of ionogel samples without the electrodes. A finest roughness of 15 ± 3 nm was observed while scanning the samples with Au cluster-assembled electrodes (Figure 3e), which is due to the organization of the gold clusters on the ionogel. No significant differences were spotted in the morphology of the samples with different amounts of ionic liquid.

### 3.2. Electrochemical Characterization

**Figure 4:**
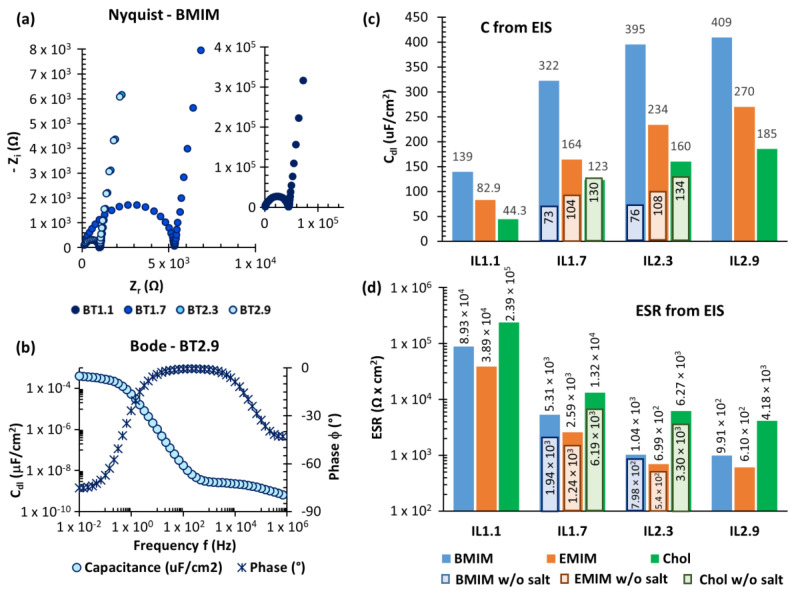
(**a**) Nyquist plots of the samples with the BMIM(TFSI) ionic liquid; (**b**) Bode plot of the sample BT2.9, showing the double layer capacitance and the current phase according to the frequency of the applied AC; (**c**,**d**) capacitance and equivalent series resistance (ESR) (respectively) of all tested supercapacitors, calculated from Electrochemical impedance spectroscopy (EIS) characterization.

Initially, all samples underwent electronic impedance spectroscopy in ambient conditions in order to obtain a preliminary screening on their double layer capacitance C_dl_ and equivalent series resistance ESR (directly related to their maximum stored energy and power delivery, respectively). Figure 4a reports the Nyquist plots obtained from samples with the BMIM(TFSI) ionic liquid. At high frequencies, we observe a semicircle typical of RC (resistor-capacitor) series, due to the contribution of ESR and the dielectric capacitance (present in any solid electrolyte). The vertical trend at lower frequencies is associated with the C_dl_ typical of supercapacitors, that was calculated according to Equation (1). All the samples with BMIM(TFSI) resulted in curves with the same profile, but those with a higher amount of IL (ionic liquid) possessed lower impedance values (in both real and imaginary components). Figure 4b is related to the sample BT2.9 and it shows the profile of C_dl_ and the phase delay according to the applied frequency. Histograms reporting a summary of C_dl_ and ESR for all samples investigated (with different IL, as well as w/o TBAF salt) are reported in Figure 4c,d.

As expected, higher amounts of ionic liquid led to lower ESR and higher C_dl_. ESR values also varied in the range between 10^2^ and 10^4^ Ω × cm^2^, accordingly to the ionic resistivity of the employed ILs: EMIM(TFSI) < BMIM(TFSI) < Chol(TFSI). C_dl_ varied as well in a range between 73 and 134 μF/cm^2^, in relation to the size of the ionic liquids cations BMIM > EMIM > Chol: the smaller the cation, the higher the capacitance value [25]. In all the examined cases, the addition of TBAF leads to an increase of ESR, as a consequence of two possible effects. The introduction of the new ammonium cation (TBA^+^) and fluoride anion (F^−^) anion changed the crystalline/liquid equilibrium of the ionic phase resulting in a lower ionic migration. As TBA^+^ and F^−^ ions are smaller than the ions of the IL, they can penetrate inside the smaller pores of the electrodes, leading to an increase of ESR. The small size of TBAF ions also resulted in a higher double-layer capacitance C_dl_, especially for the samples with BMIM(TFSI) ionic liquid, reaching maximum values of about 400 μF/cm^2^. The same EIS analysis was also carried out on all the samples in inert nitrogen atmosphere and no differences were spotted. As expected, the electrochemical behaviour of these bioplastic based EDLCs showed to be unaffected by environmental humidity.

**Figure 5:**
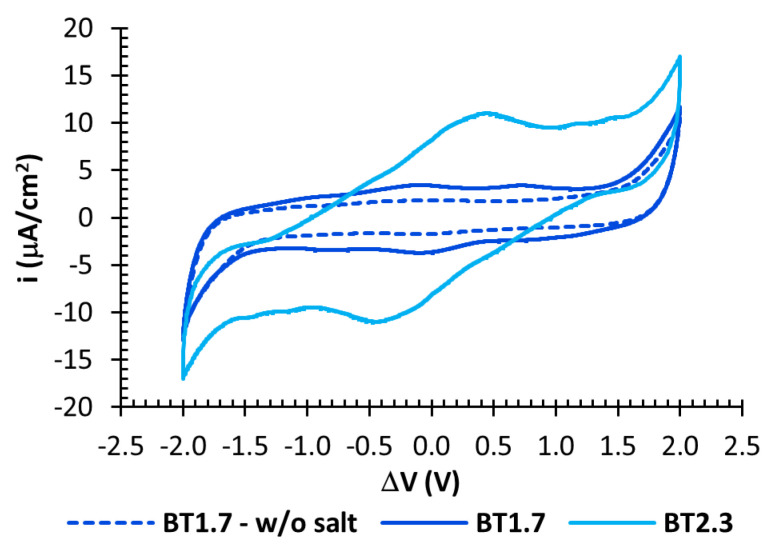
Cyclic voltammetry of the samples with BMIM(TFSI) ionic liquid, collected at 10 mV/s.

Cyclic voltammetry (CV) was then carried out on all samples to detect their window of electrochemical stability. Figure 5 reports the curves obtained from representative samples with BMIM(TFSI) ionic liquid. All of them showed electrochemical reactions of the ionogel with an applied potential difference of about 2 V, implying that they cannot operate like supercapacitors at higher potentials. The sample BT1.7 w/o the organic salt (dashed line) showed the flattest capacitive window between ±1.5 V, while the addition of TBAF led to the presence of small broad peaks at low applied potential differences. They are likely due to fast electrochemical redox or adsorption involving the ions of TBAF and the ionogel-electrode interface. As can be seen, the larger the salt amount, the highest the peaks. Nevertheless, these phenomena did not prevent the overall capacitive behaviour of the supercapacitors with TBAF, as it can be seen below. CV was also carried out on the samples with the EMIM(TFSI) and Chol(TFSI) ionic liquids, showing a similar behaviour. The experiments were repeated in inert nitrogen atmosphere and no differences were found. A representative plot is reported in the Supplementary Materials (Figure S2).

**Figure 6:**
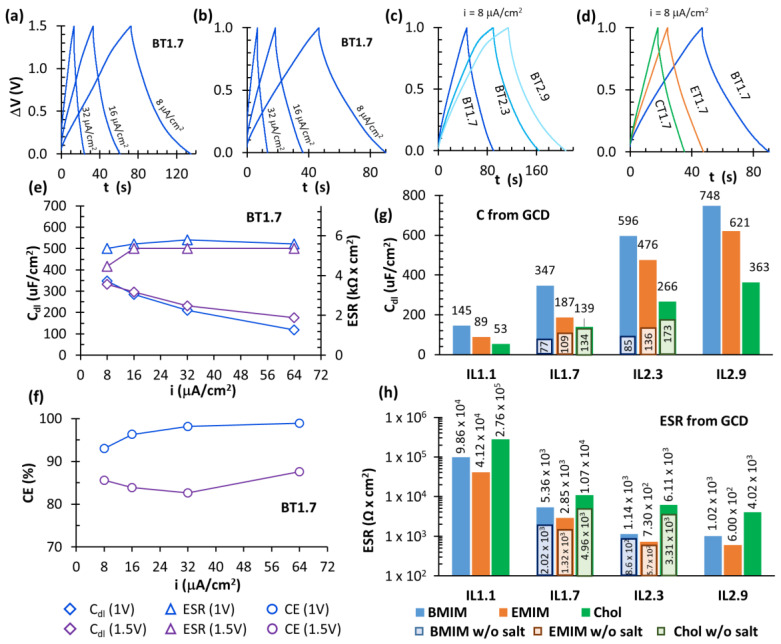
Galvanostatic charge and discharge (GCD) curves of: (**a**,**b**) BT1.7 at i_A_ = 8, 16, 32 μA/cm^2^ and ∆V_max_ =1.5 V and 1 V, respectively; (**c**) BT at different concentration at i_A_ = 8 μA/cm^2^ and ∆V_max_ = 1 V; (**d**) different ILs at the same concentration (1.7), i_A_ = 8 μA/cm^2^ and ∆V_max_ = 1V. (**e**,**f**) C_dl_, ESR and coulombic efficiency (CE) calculated for BT1.7 at different applied currents; (**g**,**h**) Capacitance and ESR (respectively) of all supercapacitors, calculated from GCD characterization (∆V_max_ = 1.0 V and i_A_ = 8 μA/cm^2^).

Galvanostatic charge and discharge (GCD) experiments were then carried out to test supercapacitor’s performances in a real operating regime. Figure 6a,b show the GCD curves of the sample BT1.7, with an applied current densities *i_A_* of 8, 16 and 32 μA/cm^2^ and up to a maximum potential V_max_ of 1.5 V and 1.0 V. The traditional triangular shape was observed for both the value of ∆V_max_; as expected, the time required for charge ∆t_c_ and discharge ∆t_d_ showed to increase for higher values of ∆V_max_ and i_A_. The values of C_dl_, ESR, and coulombic efficiency (CE) were calculated from the curves according to Equations (2) and (3), and they are reported in the graphs in Figure 6e,f. It can be seen how the capacitance increased for lower applied currents while the ESR was more constant. Working at 1.0 V or 1.5 V did not significantly change their values. The coulombic efficiency at 1.0 V had an average value of 96.5% while it decreased to 85.0% at 1.5 V. We assumed that small non-reversible redox processes occurred between 1.0 and 1.5 V, reducing the coulombic efficiency but not preventing the supercapacitors to properly work.

The same GCD characterization was carried out on all the other samples. Figure 6c reports the curves of supercapacitors with BMIM(TFSI) as ionic liquid at different concentrations (∆V_max_ = 1.0 V and i_A_ = 8 μA/cm^2^). From the graphs, it is clear that the samples with more ionic liquid took more time to charge and discharge and they can store a higher amount of energy. Figure 6d reports a comparison of GCD plots of samples with different IL but at the same concentration. The overall values of C_dl_ and ESR of all the samples, calculated from GCD plots, are coherent with those obtained through EIS measurements and are reported in Figure 6g,h.

**Figure 7:**
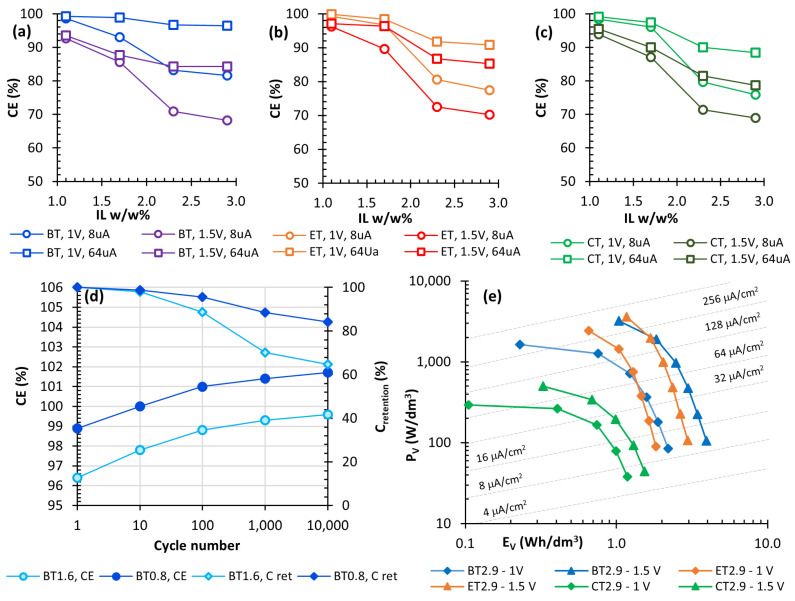
(**a**–**c**) CE values extracted from GCD curves with ∆V_max_ = 1.0 and 1.5 V and i_A_ = 8 and 64 μA/cm^2^ as function of IL concentration; (**d**) CE and C_retention_ values tested under 1 × 10^4^ working cycles (∆V_max_ = 1.0 V and i_A_ = 64 μA/cm^2^); (**e**) Ragone plot reporting the volumetric energy E_V_ and the average delivery power P_V_ obtained from the samples with the higher loading of ionic liquid (BT2.9, ET2.9, CT2.9). All measurements have been taken in air environment.

Figure 7a–c reports the calculated CE values at 8 and 64 μA/cm^2^ up to ∆V_max_ of 1.0 and 1.5 V. At higher applied currents, samples with a low amount of ionic liquid showed CE values almost always higher than 90%. This value decreased for ∆V_max_ = 1.5 V and lower currents, confirming that the ionic liquid is involved in small redox processes occurring near 1.5 V, causing some irreversible capacity loss. Consistently, this phenomenon is more pronounced for those samples with higher loadings of ionic liquid, leading to lower values of CE, down to 70% ca. This behaviour was the same regardless the type of IL. In this case too, GCD analyses were repeated in an inert atmosphere of N_2_ and no significant differences were observed. The cyclic stability of samples BT1.7 and BT2.9 was also tested (Figure 7d). After 10^4^ cycles, the capacitance retention dropped to 85% for BT1.7 and to 65% for BT2.9. This can be explained by assuming a slow electrochemical degradation of the ions, as also observed by cyclic voltammetry. Instead, the coulombic efficiency increased, slightly surpassing the original value (CE > 100.0%), probably due to a gradual rearrangement of the soft ionogel-Au clusters interface. The maximum storable energy E and the average delivery power P of EDLCs were calculated and normalized per the electrode’s volume (according to Equations (4) and (5)), since volumetric hindrance is of primary importance for microelectronics applications. Figure 7e shows a typical Ragone plot that reports E_V_ and P_V_ values for the supercapacitors with the highest loading of ionic liquid. The different formulations cover a wide area with energies ranging from about 0.2 to 4 Wh/dm^3^ and powers from 38 W/dm^3^ to 3.6 kW/dm^3^. As expected, the values increased when ∆V_max_ passed from 1.0 to 1.5 V. Consistently with previous measurements, the samples with BMIM(TFSI) can store the higher amount of energy, while those with EMIM(TFSI) are the more suited to operate in a high-power regime.

The results of the electrochemical characterization show that a bioplastic polymer like PHB can be blended with different ionic liquids to develop EDLC devices, able to stably operate at 1.5 V for 10^4^ cycle without being affected by environmental humidity. By varying the amount and the type of the IL, the properties and the performances of the supercapacitors can be tuned over a wide range. In particular, the BMIM(TFSI)-TBAF ionic mixture was the one able to provide the higher C_dl_ (750 μF/cm^2^), while the EMIM(TFSI) conferred the lowest ESR (ca. 600 Ω × cm^2^). Chol(TFSI) was instead the ionic liquid that allowed to obtain the safer ionogels thanks to the natural origin of the choline cation. The obtained energy and average delivery power densities, up to 4 Wh/dm^3^ and 3.6 kW/dm^3^ are comparable or even better than those of many ionogel-based supercapacitors developed in recent years. As an example, similar ionic liquids have been employed to develop solid supercapacitors by Tiruye et al. [54] and Gunday et al [58]. In their works, the electrodes are mainly composed by extremely light active carbon and they could reach high values of specific stored energy (10–40 Wh/kg) and delivery power (ca. 1 kW/kg). But considering the volume of the electrodes instead of their mass, the volumetric energy and power result to be ca. 10 Wh/dm^3^ and ca. 0.4 kW/dm^3^, respectively. Pandey et al. [59] and Ortega et al. [60] also reported about supercapacitors obtained with imidazolium-based ionic liquids and multi-walled carbon nanotubes electrodes, characterized by specific stored energies of 20–30 Wh/kg and delivery powers of 1–20 kW/kg. Their electrodes need to be coupled with conductive metallic foils of aluminum or graphite and then the volumetric energy and power values drop down to 1–6 Wh/dm^3^ and 0.2–0.9 kW/dm^3^, respectively. Moreover, they were obtained with ionogels based on polyvinilidene fluoride (PVDF), a non-degradable synthetic polymer, processed with pollutant organic solvents.

The supercapacitors reported in this work are based instead on natural-derived biodegradable PHB and the cluster-assembled Au electrodes are deposited directly on the surface of the ionogels, without the need to be coupled with further conductive support electrodes. Moreover, acetic acid is the only solvent employed in the whole fabrication process. Concerning the energetic performances, further improvements could be reached with the integration of high-surface area carbon-based electrodes. For example, thin and highly porous carbon films can be deposited on the surface of the ionogels by means of supersonic cluster beam deposition technique [61]. Active carbon and carbon nanotubes could also be employed: they can be added to the PHB-IL mixture and casted from acetic acid to form bucky gel electrodes [62].

## 4. Conclusions

This work demonstrates that solid supercapacitors based on biodegradable natural-derived linear polyester as the poly(3-hydroxybutyrrate) PHB can be produced by a facile and fast fabrication process, blending the polymer with different ionic liquids and providing thin Au electrodes with SCBD technique. The developed devices are resistant to environmental humidity, suggesting that they can operate in various environments without encapsulation. Moreover, the electrochemical analyses allowed to deeply understand the role and the effect of the different amount and type of ionic liquid, as well as TBAF. Based on a biodegradable polymer, these findings represent an innovative solution in the field of eco-friendly organic electronics with a potential focus for mobile and portable electronics industries, where compact devices, rather than lightweight, are the preferred choice. Novel applications of this type are being currently developed in our laboratories [63].

## 5. Patents

Flexible, biodegradable, and biocompatible supercondenser. P. Saettone, G. Generali, T. Santaniello, L. Migliorini, P. Milani, M. Cifelli, I. Monaco, M. Comes Franchini. WO2020109841 (A1). Applicant Bio-On spa.

## Supplementary Materials

The following are available online at https://www.mdpi.com/2079-4991/10/10/2062/s1, Table S1: Ionogels formulation, Figure S1: setup for electrochemistry, Figure S2: CV in inert atmosphere.

## Appendix A

### Experimental Details on AFM Measurements

The morphology of the ionogels was characterized by Atomic Force Microscopy (AFM) using a Bioscope Catalyst (Bruker) instrument, operated in Peak-Force Tapping Mode in air; it is equipped with silicon nitride cantilevers mounting single crystal silicon tips (with nominal radius of 8–12 nm), characterized by a resonance frequency in the range 50–90 kHz and force constant k = 0.4 N/m. Several images were acquired with a scan rate of 0.5 Hz and sampling resolution of ~1 nm/pixel, in order to characterize the morphology of the interface. The images were flattened by line-by-line subtraction of first and second order polynomials in order to remove artifacts due to sample tilt and scanner bow. From flattened AFM images, the root-mean-square surface roughness R_q_ was calculated as the standard deviation of surface heights. The correlation length ξ, i.e., the characteristic length over which two randomly chosen points on the surface have uncorrelated heights, is calculated as the decay length of the height correlation function; ξ represents a statistical estimation of the width of the largest surface pores.
