# Supplementary material for: Eco-Friendly Supercapacitors Based on Biodegradable Poly(3-Hydroxy-Butyrate) and Ionic Liquids
Source: Nanomaterials (Basel). 2020 Oct 19;10(10):2062. doi: 10.3390/nano10102062 (PMC7603249; doi:10.3390/nano10102062)
Supplement: Supplementary file 1 [file nanomaterials-10-02062-s001.pdf]

nanomaterials

Supplementary Materials

Eco-Friendly Supercapacitors Based on Biodegradable Poly(3-Hydroxy-Butyrate) and Ionic Liquids

Lorenzo Migliorini [1], Tommaso Santaniello [1], Francesca Borghi [1], Paolo Saettone [2], Mauro Comes Franchini [2,3,*], Gianluca Generali [2] and Paolo Milani [1,*]

1 Interdisciplinary Centre for Nanostructured Materials and Interfaces (CIMaINa), Physics Department, University of Milan, Milano, 20133, Italy; lorenzo.migliorini@unimi.it (L.M.); tommaso.santaniello@unimi.it (T.S.); francesca.borghi@unimi.it (F.B.)
2 Bio-On spa, Via Santa Margherita al Colle 10/3 40136, Bologna, Italy; Paolosaettone@yahoo.it (P.S.); gianluca.generali@bio-on.it (G.G.)
3 Department of Industrial Chemistry "Toso Montanari", University of Bologna, Viale Risorgimento 4, 40136 Bologna, Italy
* Correspondence: mauro.comesfranchini@unibo.it (M.C.F.); paolo.milani@mi.infn.it (P.M.)

Table S1. Ionogels formulation

SAMPLE	IL	mg of IL/mg of PHB	TBAF
BT1.1	BMIM(TFSI)	1.1	yes
BT1.7	BMIM(TFSI)	1.7	yes
BT1.7 w/o salt	BMIM(TFSI)	1.7	no
BT2.3	BMIM(TFSI)	2.3	yes
BT2.3 w/o salt	BMIM(TFSI)	2.3	no
BT2.9	BMIM(TFSI)	2.9	yes
ET1.1	EMIM(TFSI)	1.1	yes
ET1.7	EMIM(TFSI)	1.7	yes
ET1.7 w/o salt	EMIM(TFSI)	1.7	no
ET2.3	EMIM(TFSI)	2.3	yes
ET2.3 w/o salt	EMIM(TFSI)	2.3	no
ET2.9	EMIM(TFSI)	2.9	yes
CT1.1	Chol(TFSI)	1.1	yes
CT1.7	Chol(TFSI)	1.7	yes
CT1.7 w/o salt	Chol(TFSI)	1.7	no
CT2.3	Chol(TFSI)	2.3	yes
CT2.3 w/o salt	Chol(TFSI)	2.3	no
CT2.9	Chol(TFSI)	2.9	yes

Table S1 reports the detailed formulation of the synthesized ionogels. For all of them, PHB powder was dissolved in acetic acid with a concentration of 50 mg/mL. Different types and amounts of ionic liquid were added to the mixture according to the values reported in the table. In some cases, TBAF was previously dissolved in the ionic liquid at a concentration of 6.85% *w*/*w*.

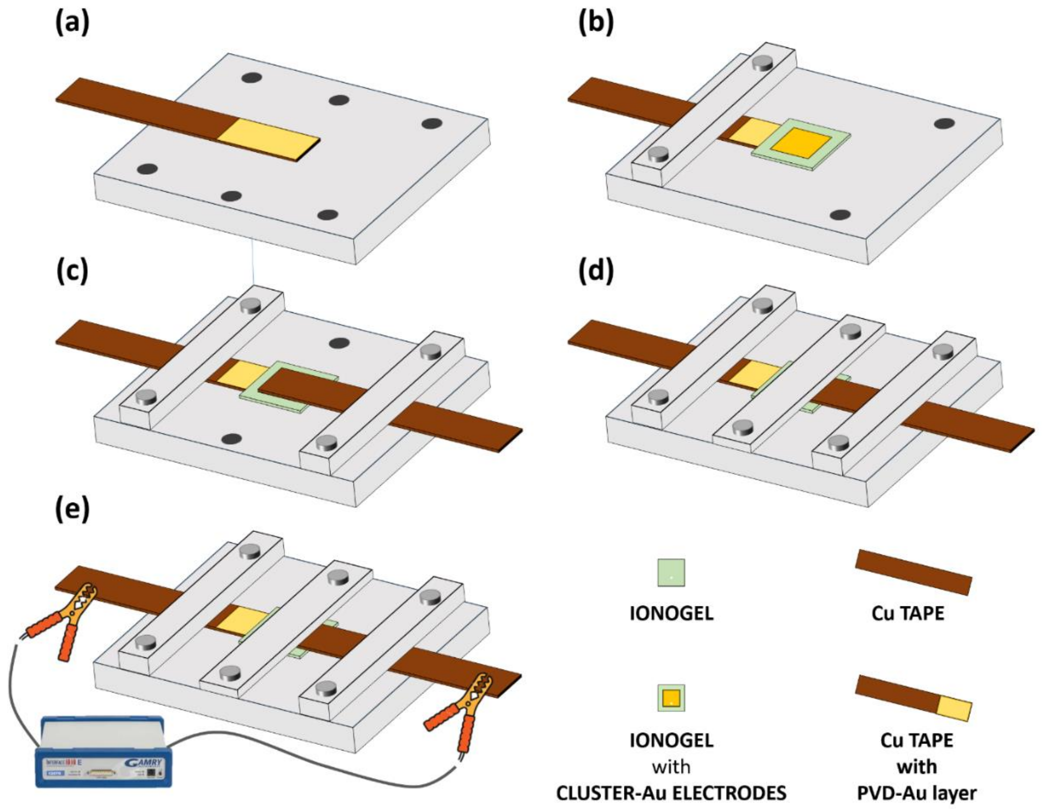

Figure S1. Setup for electrochemical characterizations

Figure S1 shows the experimental setup employed for the two-electrodes electrochemical characterizations. Physical vapour deposition was used to deposit a thin layer of gold on the non-sticky surface of copper tape strips that were then put in contact with the supercapacitor's electrodes.

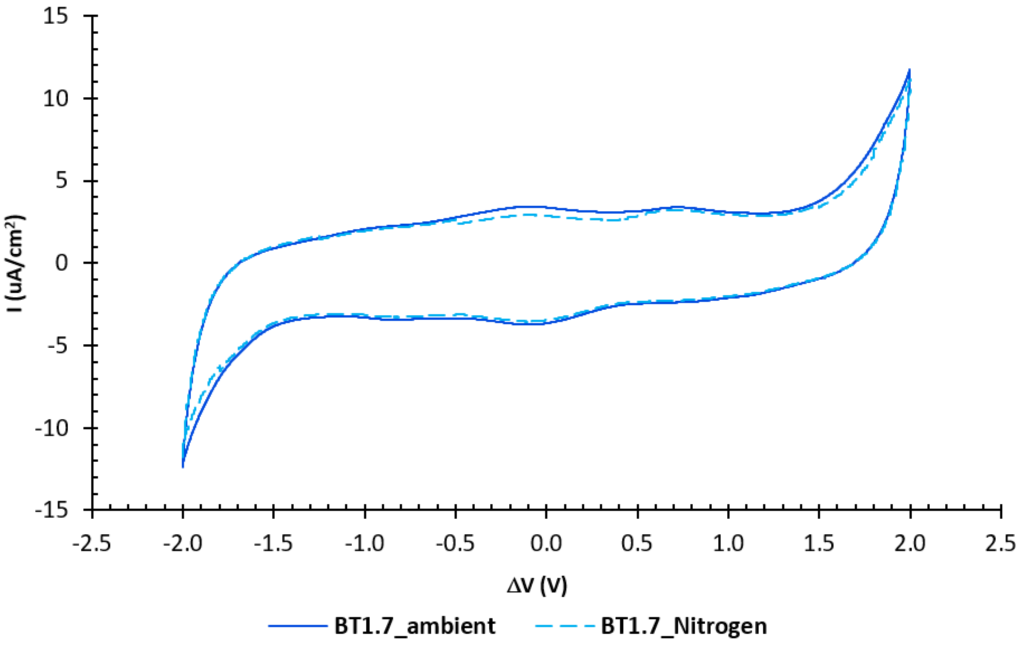

Figure S2. Cyclic voltammetry in ambient conditions and in inert nitrogen atmosphere

The graph reports two cyclic voltammetries (CVs) carried out on the sample BT1.7 (scan rate of 10 mV/s). One CV was conducted in ambient conditions while the other in inert nitrogen atmosphere inside a glove box. As can be seen, the two voltammograms are not significantly different.

© 2020 by the authors. Submitted for possible open access publication under the terms and conditions of the Creative Commons Attribution (CC BY) license (http://creativecommons.org/licenses/by/4.0/).
